# Peripheral quantitative computed tomography and blood biomarkers in children with spinal cord disorders

**DOI:** 10.1038/s41394-025-00720-2

**Published:** 2025-08-20

**Authors:** Jamie Ellis, Mary P. Galea, Adam Scheinberg, Peter Simm

**Affiliations:** 1 Murdoch Children’s Research Institute, Parkville, VIC Australia; 2 Department of Medicine (Royal Melbourne Hospital), The University of Melbourne, Parkville, VIC Australia; 3 Department of Paediatrics, The University of Melbourne, Melbourne, VIC Australia; 4 Victorian Paediatric Rehabilitation Service, The Royal Children’s Hospital, Melbourne, VIC Australia; 5 Department of Endocrinology and Diabetes, The Royal Children’s Hospital, Melbourne, VIC Australia

**Keywords:** Paediatrics, Spinal cord diseases, Bone

## Abstract

**Study design:**

Cross-sectional study

**Objectives:**

Spinal cord disorders (SCD) in children are uncommon, but for those affected the musculoskeletal effects can be severe. Little is known about bone health and pediatric SCD experiences in Australia. We aimed to describe bone and muscle development following pediatric SCD.

**Setting:**

The Royal Children’s Hospital, Melbourne, Australia

**Methods:**

Ten participants with SCD were recruited and underwent peripheral quantitative computed tomography (pQCT) scans and blood tests to observe bone health biochemistry.

**Results:**

Z-scores (mean ± SD) for trabecular density at the 4% tibial site were lower in non-weightbearing children compared to weightbearing children (−6.5 ± 1.5 vs. −2.4 ± 1.5, Total cohort: −5.0 ± 2.6). At the 66% site, muscle cross-sectional area (−4.7 ± 2.2 vs. −1.1 ± 1.7, Total cohort: −3.1 ± 2.7), strength strain index (−3.4 ± 1.3 vs. −1.0 ± 0.4, Total cohort: −2.5 ± 1.6) and total bone cross-sectional area (−2.4 ± 0.8 vs. 0.4 ± 1.7, Total cohort: −1.2 ± 1.9) were also lower in non-weightbearing children. Radial Z-scores revealed reduced total bone area at the 4% site (−3.5 ± 2.1) and strength strain index at the 65% site (−1.3 ± 1.8) in all participants. Serum testing revealed alkaline phosphatase was reduced in three participants, one of whom was also deficient in phosphate and 25-Hydroxyvitamin D.

**Conclusions:**

Weightbearing status influenced multiple outcomes including trabecular density, muscle cross-sectional area and bone strength in the tibia.

## Introduction

Spinal cord disorders (SCD) in children are uncommon but those affected can suffer severe musculoskeletal consequences. Because children have a developing skeleton, the effects of injury can differ significantly from those seen in adults with SCD [1]. The loss of neural input and compromised musculoskeletal interaction causes the disruption of bone growth below the injury site [2, 3]. This poses significant implications for later life. Despite the significant negative effect of SCD on bone health in children, there is a lack of evidence-based clinical practice guidelines (CPGs) for treatment of bone health.

Following SCD in both children and adults, bone health parameters such as bone mineral content, bone mineral density (BMD), cross-sectional area (CSA), geometry, and bending strength, as measured by the polar strength strain index (pSSI), can be severely compromised [4, 5]. Lean tissue physiology is also altered, resulting in extensive muscle atrophy [6]. Muscle and bone are tightly interlinked systems, having been described as “the muscle-bone unit,” whereby tensile forces from the muscle act on bone to influence parameters such as mass and strength [7]. As such, while these tissues influence each other in terms of growth, they also deteriorate synchronously [8]. When muscle loses neural input and can no longer exert tensile forces upon bone, there is rapid decrease in bone size and strength [8]. Bone health is an extremely important consideration for patients with SCD as it can underscore a myriad of other complications, including bony pathologies like scoliosis or fractures, thus increasing the pain experience, and decreasing quality of life [9, 10].

While scoliosis and hip dysplasia are common musculoskeletal issues in pediatric SCD that are confirmed radiographically [11, 12], bone health as measured by BMD in children with SCD has predominantly been assessed using dual-energy X-Ray absorptiometry (DXA) [13–15]. However, there are no CPGs designed for the specific monitoring or treatment of bone health issues using DXA in children with SCD in Australia.

Peripheral Quantitative Computed Tomography (pQCT) is used to scan peripheral areas of the body such as the distal tibia and radius, revealing parameters of bone that include volumetric BMD, muscle density, and bone geometry [16]. It is also able to assess trabecular bone and cortical bone independently, unlike DXA scans [17]. PQCT is an alternative imaging modality to DXA that could be utilized in this population to assess bone health, and it may be a superior method due to its ability to measure several bone parameters with higher resolution than DXA [16, 17], however, there is currently very little data on bone health in pediatric SCD using pQCT imaging [5, 18], and it is not used routinely in clinical practice. Only one study has attempted to explore its clinical utility in a pediatric SCD cohort in Australia, which revealed decreased trabecular density, cortical cross-sectional area and bone strength, and increased circularity, of the tibia in these children [5].

Blood biomarkers also provide valuable insight to the status of bone health for children with SCD. Calcium, vitamin D, alkaline phosphatase (ALP) and phosphate are important elements in maintaining the health of bone. Following SCD, vitamin D levels have been observed to decrease significantly [19], and serum calcium levels can increase which may indicate liberation of calcium from bone tissue, an effect of immobility, thus contributing to bone deterioration [3]. Phosphate is important for bone growth and mineralization, and ALP is an enzyme that liberates phosphate for bone formation activity [20]; therefore, they are common markers of bone turnover [21]. However, CPGs utilizing blood biomarkers for bone health monitoring in this population are also lacking.

The aim of this study was to observe how bone and muscle development are altered by pediatric SCD. We hypothesized that lower extremity bone would deteriorate as a function of weightbearing status in children with SCD, with greater reductions in leg BMD, pSSI and muscle CSA in non-ambulant children.

## Methods

### Participants and procedure

Potential participants were identified through electronic medical record search or through clinician recruitment at The Royal Children’s Hospital. Participants who consented to the study underwent pQCT scans for musculoskeletal health outcomes, and blood tests for biomarkers of bone health.

Inclusion Criteria

1. Children under the age of 21 years at time of recruitment, and between the ages of 0 and 18 years at time of SCD diagnosis, who received treatment at The Royal Children’s Hospital, Melbourne, Australia.
2. SCD diagnosis classified according to the International Statistical Classification of Diseases and Related Health Problems, 10^th^ Revision, Australian Modification [22] diagnostic codes relating to Traumatic, Non-traumatic, nonspecific lesions for acute paraplegia and tetraplegia, Cauda Equina syndrome and other SCD-related codes.

Exclusion Criteria

1. Children who had a primary code not included in the above list, i.e., multiple sclerosis (G35) or neural tube defects (e.g., spina bifida)
2. Existence of premorbid neuromuscular condition or known primary bone disorder

### Instruments

#### Medical history

Participant demographic and injury-related information were obtained from the electronic medical record of The Royal Children’s Hospital. This information included date of birth, date of diagnosis, injury level, injury severity and etiology. Injury Severity was defined using the American Spinal Injury Association Impairment Scale (AIS) Level, which was classified using the International Standards for Neurological Classification of Spinal Cord Injury Worksheet.

#### Musculoskeletal assessment

BMD and associated parameters of the radius and tibia were measured with pQCT using a Stratec XCT-2000 (Stratec Inc., Pforzheim, Germany). The sites investigated were the 4 and 65% radius, and 4 and 66% tibia. Scans were performed and analysed, and conversion from pQCT raw data to age-matched Z-scores, based on published pediatric reference data [23–25], however tibia Z-scores could not be generated for one participant due to lack of age-appropriate reference data. The bone and muscle parameters assessed using pQCT at the 4% site of the radius were total bone CSA, trabecular volumetric BMD, and total volumetric BMD. Cortical volumetric BMD, total bone CSA and muscle area of the radius were assessed at the 65% site. Trabecular density was measured at the 4% tibial site, with cortical density, cortical CSA, total bone CSA, pSSI and muscle CSA being assessed at the 66% site of the tibia.

#### Serum tests

Blood tests were performed by phlebotomists at The Royal Children’s Hospital, and levels of calcium, phosphate, vitamin D and ALP were analyzed as indicators of bone health.

### Analysis

Due to the small study sample, descriptive statistics were used to present all data. Results are reported as mean ± standard deviation unless otherwise specified. All analyses were performed using Microsoft Excel 2024.

### Statement of ethics

This study was approved by The Royal Children’s Hospital Melbourne Human Research Ethics Committee (QA81147) and informed consent was obtained from all participants and parents before participation. All methods were performed in accordance with the relevant national and local guidelines and regulations.

## Results

Ten children with SCD were enrolled in this study (Table 1). All participants received blood tests. Two participants were unable to undergo pQCT at the tibia due to limb contractures, but one of these participants was able to undergo pQCT at the radius.

**Table 1:** Participant demographics and injury characteristics.

Participant	Sex	Age (Years)	Age at injury (Years)	Injury Level	Injury Severity (AIS Level)	Injury etiology	Mobility status
1	Female	8	4	C4/5	A	MVA	Power wheelchair
2	Male	13	13	C5	A	Sport - bicycle accident	Power wheelchair
3	Male	18	6	C2/3	D	MVA	Ambulant
4	Male	17	15	C4	B	MVA	Power wheelchair
5	Female	13	13	T11	D	Transverse myelitis	Ambulant
6	Male	10	6	T6/T7	B	Acute necrotizing myelitis	Manual wheelchair
7	Female	20	12	T7	D	Iatrogenic - scoliosis surgery	Ambulant
8	Female	13	13	T9/T10	C	Central cord syndrome	Ambulant with aids
9	Male	15	Birth	T12	C	Lumbar puncture	Manual wheelchair
10	Male	18	3	T4	A^a^	MVA	Manual wheelchair

Injury Severity was defined using the American Spinal Injury Association Impairment Scale (AIS) Level, which was classified using the International Standards for Neurological Classification of Spinal Cord Injury Worksheet.

^a^Participant 10 did not receive a formal AIS classification but had a complete motor/sensory injury based on clinical examination.

As presented in Table 2, pQCT assessment revealed that the mean ± SD of several age-matched Z-scores trended towards a decrease in non-weightbearing children compared to weightbearing children, including trabecular density (−6.5 ± 1.5 vs. −2.4 ± 1.5) at the 4% site of the tibia, and muscle CSA (−4.7 ± 2.2 vs. −1.1 ± 1.7), strength strain index (−3.4 ± 1.3 vs. −1.0 ± 0.4) and total bone CSA (−2.4 ± 0.8 vs. 0.4 ± 1.7) at the 66% site. At the 4% site of the radius, the mean ± SD of total bone CSA decreased across all participants (−3.5 ± 2.1). At the 65% site, the mean ± SD of cortical density was reduced in non-weightbearing children (−2.1 ± 3.2 vs. 1.3 ± 1.0).

**Table 2:** Z-scores of bone and muscle parameters measured using peripheral quantitative computed tomography.

	Total	Non-weightbearing	Weightbearing
**TIBIA**			
*4% site*			
Trabecular density	−5.0 ± 2.6	−6.5 ± 1.5	−2.4 ± 1.5
*66% site*			
Cortical density	0.7 ± 4.7	0.5 ± 6.2	1.1 ± 0.3
Total CSA	−1.2 ± 1.9	−2.4 ± 0.8	0.4 ± 1.7
Cortical CSA	−1.4 ± 2.6	−2.7 ± 1.0	0.3 ± 3.3
Muscle CSA	−3.1 ± 2.7	−4.7 ± 2.2	−1.1 ± 1.7
pSSI	−2.5 ± 1.6	−3.4 ± 1.3	−1.0 ± 0.4
**RADIUS**			
*4% site*			
Total CSA	−3.5 ± 2.1	−3.7 ± 1.3	−3.3 ± 2.6
Trabecular density	−1.3 ± 2.8	−2.1 ± 3.3	−0.2 ± 2.2
Total density	−0.3 ± 1.2	−0.8 ± 1.3	0.1 ± 1.1
*65% site*			
Cortical density	−0.9 ± 3.0	−2.1 ± 3.2	1.3 ± 1.0
Total CSA	2.1 ± 1.5	2.2 ± 1.1	2.1 ± 2.2
Muscle CSA	−0.3 ± 2.6	−0.1 ± 2.5	−0.6 ± 3.4
pSSI	−1.3 ± 1.8	−1.2 ± 2.0	−1.5 ± 1.8

Data presented as mean ± standard deviation. N = 8–9.

**Fig. 1:**
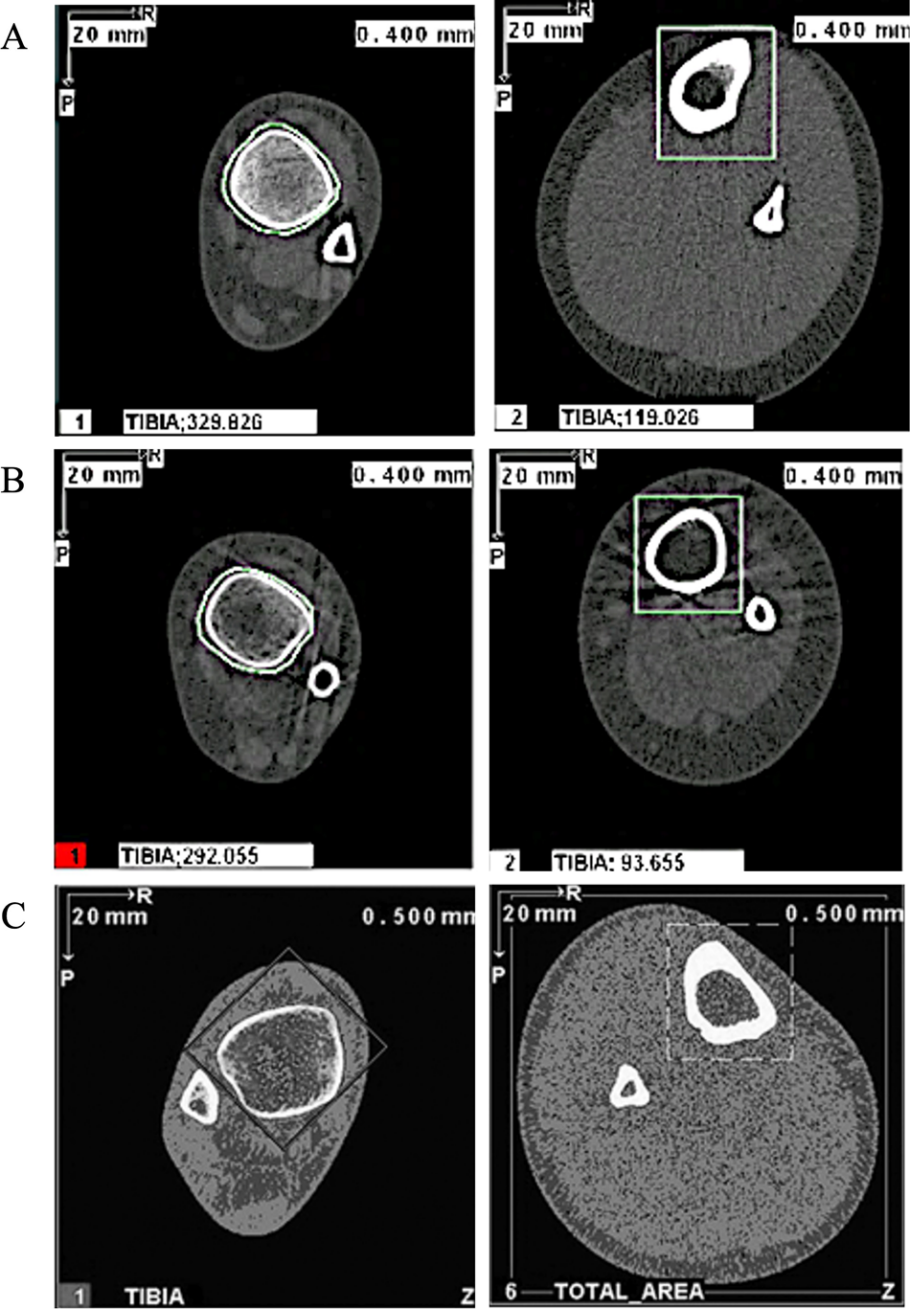
Geometrical Changes of the Tibia. Cross-sectional view of the tibia at the 4% (left) and 66% (right) sites, showing bone geometry in (**A**) weightbearing child with SCD, (**B**) non-weightbearing child with SCD, and (**C**) healthy control (from Adams et al [26]) measured by peripheral quantitative computed tomography. Panels A and B were representative images selected from weightbearing and non-weightbearing children, participants 5 and 6, respectively.

Figure 1 illustrates the geometry changes that occur at the tibia in children with SCD who are weightbearing (A) or non-weightbearing (B), with reference to a healthy control (C) [26]. Fig. 1A depicts the tibia at the 66% site (right image) of a weightbearing child with SCD, which can be observed to have a more ‘teardrop’ shape than the tibia of a non-weightbearing child in Fig. 1B, which is more circular and has thinner cortical bone.

Serum testing for bone health markers included calcium, phosphate, ALP and 25-Hydroxyvitamin D. Of the ten participants tested, the mean ± SD of ALP were lower in three participants (61.3 ± 9.7 U/L, reference range 100–350 U/L). ALP reference ranges differ according to age, but these three participants were of similar age and this reference range presented is applicable for these children. All other participants were within their respective reference range for ALP. Two of these three children were non-weightbearing. One of these children also had reduced phosphate (0.98 mmol/L, reference range 1.10–1.90 mmol/L), and was insufficient in 25-Hydroxyvitamin D (48 nmol/L, reference range >50 nmol/L). This child was a non-weightbearing participant.

## Discussion

We report pQCT and biochemical outcomes from a study that explored a range of outcomes relating to SCD in children. We hypothesized that weightbearing ability would influence the bone parameters measured by pQCT such as bone mineral density, muscle cross sectional area, bending strength and geometry. While our sample size was not large enough to yield data with sufficient power to address this hypothesis, we observed trends towards several clinically relevant changes related to weightbearing ability. Additionally, we reported on biochemical changes related to bone health.

Overall, pQCT assessment revealed that children with SCD have trends towards lower trabecular density, total bone CSA, cortical bone CSA, pSSI and muscle CSA at the tibia, and total bone CSA, trabecular density and pSSI at the radius compared to age-matched children [23–25]. Trabecular density was lower at the tibia for all participants compared to age-matched controls, with trends towards further decreases in children who were non-weightbearing. Cortical density was similar to controls, but total bone CSA, cortical bone CSA, bending strength and muscle CSA appeared to reduce in a similar fashion to trabecular density, with a trend to further reductions in non-weightbearing children. This was expected, as previous studies in adults [4] and children [5, 14] have also established that musculoskeletal parameters in the lower limb are significantly affected by spinal cord injury and the subsequent paralysis.

Studies focusing on bone health in children with SCD are lacking, making our prospective study an important contribution to the literature. Our findings are congruent with the only other study using pQCT in children with SCD [5]. They observed reductions of a similar magnitude of trabecular density at the radius and total CSA, and similarly unchanged Z-score for cortical density, in their tetraplegic participants, compared to our non-weightbearing participants. However, while there were no differences in total CSA at the radius in their paraplegic participants compared to controls [5], it was reduced in our cohort regardless of weightbearing ability, level of injury or preservation of upper limb function.

At the tibia, trabecular density alongside total CSA, cortical CSA, muscle CSA and pSSI were also reduced, while cortical density was comparable to that of age-matched controls. This is important because our study sample was smaller than that previously published [5], however the observations are comparable, so we are confident that our findings can contribute meaningfully and demonstrate reproducibility to the scarce literature in this area. Cortical density at the tibia remained comparable to that of age-matched controls most likely due to the slower rate of bone turnover of cortical bone versus trabecular bone, which is more metabolically active [27]. Cortical bone has been found to be more resistant to losses in density compared to trabecular bone following SCD, therefore when mechanical unloading of bone occurs rapidly such as following SCD, trabecular bone is affected more than cortical bone [4, 5, 27].

Bone area, reported here as total bone and cortical bone CSA, has been observed to correlate with muscle mass and tensile muscle forces in both children with bone diseases and adults with spinal cord injury [6, 7, 28]. Therefore, it was hypothesized that tibial CSA would be reduced in children with SCD who can no longer walk. Indeed, this was observed in our data. Not only were total CSA and cortical CSA of the tibia reduced compared to age-matched controls for all participants, but there was a trend towards further reductions in non-weightbearing children. In addition, we observed bone geometry differences between weightbearing and non-weightbearing children. Taken together, the reductions in bone area and density and the changes in bone geometry, which determine the overall strength of bone [29], lead to increased fracture risk. This is a clinically relevant finding.

Muscle CSA was also observed to decrease substantially. The loss of neural input, movement and load following SCD causes the rapid atrophy of muscles, and particularly those in the lower limb [6, 30]. However, while non-weightbearing children had reduced muscle CSA, as expected, so did ambulant children. This is likely because, despite being able to bear weight and walk, they still have reduced activity compared to typically developing children.

The reductions in trabecular density, bone strength, bone and muscle CSA, and changes in geometry at the tibia that appear to be further exacerbated in non-weightbearing children are clinically relevant observations. The extent of neurological impairment determines muscle function and therefore weightbearing ability following SCD, with greater impairment resulting in less weightbearing ability [31]. This is evident in our study cohort, with the non-weightbearing children having greater impairment according to their AIS levels than the ambulant children. The subsequent loss of load through the skeleton following severe SCD seems to have significant effects on bone and muscle health and could be addressed by developing CPGs for therapies aimed at assisted standing [32] or upright posture [33].

Our pQCT data adds to the only other cohort study [5] describing how several clinically significant bone and muscle parameters change in children following an SCD diagnosis. In addition to adding more pQCT data to the literature, we complement these data by exploring blood biomarkers of bone health that have not been reported elsewhere.

Biochemical investigations of bone health markers revealed that the level of ALP was the only marker trending toward a decrease across multiple participants. This enzyme can be used as a marker of bone formation [20, 21], therefore a reduction in ALP suggests that bone turnover is reduced in these children [34]. While an exploration of therapeutic options was outside the scope of this study, it is important to consider what interventions may be possible now and in the future. The only pharmacological intervention currently used in the pediatric population are bisphosphonates, but the evidence for their effectiveness is limited [35, 36]. They have been found to reduce bone turnover and increase trabecular density [18, 37], however, with bone turnover decreasing following SCD, the lack of efficacy from bisphosphonates may be explained by this combined effect on low bone turnover rates. Future options could include anabolic therapies such as sclerostin inhibitors, which may be more effective in the context of the low bone turnover state [38]. However, despite approval for some adult indications [38, 39], pediatric trials are only just commencing in primary bone conditions [40, 41], so it will be some time before they are accessible for indications like SCD.

### Study limitations

While our findings are important, they lack sufficient power to investigate relationships between bone health parameters and important predictors, thus making generalization difficult. In our small study population, predictors such as age at injury or sex were not able to be evaluated in relation to bone and muscle changes following SCD. Due to the low prevalence of SCD, an adequately powered study would require national recruitment in our country. The only other comparable study [5] to ours was conducted in 2013 with 19 study participants, and the researchers also acknowledged their sample size as relatively small, with the need for larger studies to enable generalization of results. Given the low incidence of pediatric SCD, we recruited participants with both traumatic and non-traumatic etiologies. This may introduce further variations between participants regarding their musculoskeletal outcomes, which further affects formal analysis of these data. However, we believe it is important to include non-traumatic etiologies of acquired SCD given that they represent the majority of children with SCD.

Furthermore, due to the lack of studies reporting pQCT data, published reference datasets are lacking. While some pediatric pQCT reference datasets exist, they are not as comprehensive as those available for DXA, and they are performed in European populations which may not be fully representative of the Australian population. Also, the tibia dataset we used, which is the only pediatric one available, did not account for the young adult age range (18–21 years) encompassed by our inclusion criteria, which resulted in our inability to analyze tibial data of one of our participants. Additionally, the biomarkers analyzed here were only retrieved at a single study visit, so longitudinal monitoring is needed to ascertain exactly how the rates of bone turnover are affected following SCD diagnosis, how they are implicated in deteriorating bone health, and whether this may relate to potential interventions aimed at supporting bone health.

### Conclusion

This cross-sectional study is the second study to utilize pQCT in pediatric SCD in Australia. We observed that weightbearing is important for maintaining or at least buffering important bone and muscle parameters, so future research exploring therapies for pediatric SCD should at least include assisted-standing or upright time for non-ambulant patients. Exploring these outcomes in large, nationally coordinated longitudinal studies would help to fully understand the effects of SCD, not only on the growing musculoskeletal system, but also in allowing a broader understanding of the needs of children with SCD in Australia. Such studies would underscore ongoing evaluation for these children, and contribute to CPGs, which are needed to standardize the management of pediatric SCD. Our study highlights the need for CPGs which could include standing therapies and regular analysis of bone health biomarkers. Furthermore, increased contributions to the body of literature on pQCT in children with SCD would increase evidence for its clinical utility.

## Data Availability

The datasets generated during and/or analyzed during the current study are available from the corresponding author on reasonable request.
